# High therapeutic efficacy of artemether–lumefantrine and dihydroartemisinin–piperaquine for the treatment of uncomplicated falciparum malaria in Somalia

**DOI:** 10.1186/s12936-019-2864-1

**Published:** 2019-07-11

**Authors:** Marian Warsame, Abdillahi Mohamed Hassan, Abdikarim Hussein Hassan, Ali Mohamed Jibril, Nimol Khim, Abdulkadir Mohamed Arale, Ahamed Hassan Gomey, Zainab Said Nur, Said Mohamed Osman, Marian Said Mohamed, Ali Abdulrahman, Fahmi Essa Yusuf, Jamal Ghilan Hefzullah Amran, Benoit Witkowski, Pascal Ringwald

**Affiliations:** 1 Global Malaria Programme, World Health Organization, 20 Avenue Appia, 1211 Geneva 27, Switzerland; 2 World Health Organization, Mogadishu, Somalia; 3 Ministry of Health, Puntland, Somalia; 4 INTERSOS, Jowhar Hospital, Jowhar town, Jowhar, Somalia; 5 Malaria Molecular Epidemiology Unit, Pasteur Institute in Cambodia, Phnom Penh, Cambodia; 6 Ministry of Health and Human Service, Mogadishu, Somalia; 7 World Health Organization, Hargeisa, Somalia; 8 Malaria Translational Research Unit, Pasteur Institute, Paris, France; 9 Present Address: University of Gothenburg, Gothenburg, Sweden

**Keywords:** Artemether–lumefantrine, Dihydroartemisinin–piperaquine, *Plasmodium falciparum*, Artemisinin resistance, Piperaquine resistance, Somalia

## Abstract

**Background:**

Artemether–lumefantrine (AL) and dihydroartemisinin–piperaquine (DHA/PPQ) are the recommended first- and second-line treatments, respectively, for uncomplicated falciparum malaria in Somalia. The studies reported here were conducted to assess the efficacy of these artemisinin-based combinations and the mutations in *Plasmodium falciparum* K13-propeller (*Pfk13*) domain and amplification in *Pfplasmepsin* 2 (*Pfpm2*) gene in Somalia.

**Methods:**

One-arm prospective studies were conducted to assess the clinical and parasitological responses to DHA/PPQ and AL at two sites in 2016 and 2017, respectively, using the standard WHO protocol. The patterns of molecular markers associated with artemisinin and PPQ resistance were investigated for the first time in Somalia.

**Results:**

A total of 339 patients were enrolled with 139 for AL and 200 for DHA/PPQ. With AL, no parasite recurrence was observed among patients treated at either site, corresponding to 100% clinical and parasitological responses. For DHA–PPQ, an adequate clinical and parasitological response rate > 97% was observed. All study patients on both treatments at both sites were parasite-free on day 3. Of the 138 samples with interpretable results for the polymorphism in *Pfk13*, only one (0.7%), from Bosaso, contained a non-synonymous mutation (R622I), which is not one of the known markers of artemisinin resistance. No *Pfpm2* amplification was observed among the 135 samples with interpretable results.

**Conclusions:**

AL and DHA/PPQ were highly effective in the treatment of uncomplicated falciparum malaria, and there was no evidence of resistance to artemisinin or PPQ. These two combinations are thus relevant in the chemotherapeutic strategy for malaria control in Somalia.

*Trial registration* ACTRN12616001005448 (Jowhar DP study), ACTRN12616000553471 (Bosaso DP study), ACTRN12617001055392 (AL study in Bosaso and Jowhar)

## Background

Malaria is still a major health problem: it caused an estimated 219 million cases and 435,000 deaths worldwide in 2017, most of which occurred in Africa [1]. Artemisinin-based combination therapy (ACT) is the currently recommended anti-malarial drug for the treatment of uncomplicated *Plasmodium falciparum* [2]. Although ACT has maintained its therapeutic efficacy in most malaria-endemic countries [3], the emergence and spread of resistance to artemisinins [4–6] and, more recently, to piperaquine (PPQ) [7, 8] in South-East Asia are of great concern. Artemisinin resistance, defined as delayed parasite clearance [3], has been associated with mutations in the *P. falciparum k13*-propeller (*Pfk13*) domain [9], and PPQ resistance has been linked to *Pfplasmepsin* 2 (*Pfpm2*) gene amplification [10]. A rapid increase in the treatment failure rate with dihydroartemisinin–piperaquine (DHA/PPQ) has been reported in Cambodia, Thailand and Viet Nam [7, 8, 11–13]. Non-synonymous *Pfk13* mutations are so far rare in Africa, and the mutation most frequently observed is A578S, which is not associated with clinical or in vitro resistance to artemisinins [14, 15].

The World Health Organization (WHO) recommends routine monitoring of the efficacy of the recommended ACT at least every 2 years [16]. This is essential, particularly in view of the reported multidrug resistance to anti-malarial medicines and the declining efficacy of DHA/PPQ in South East Asia. A study of therapeutic efficacy in vivo, conducted with the standard WHO protocol [17], is the gold standard for monitoring the efficacy of anti-malarial medicines in order to guide effective malaria treatment. Analysis of molecular markers of drug resistance is an additional monitoring tool to support in vivo confirmation of parasite resistance.

The National Malaria Control Programme (NMCP) of Somalia recommended artesunate + sulfadoxine/pyrimethamine (AS + SP) as first-line treatment in 2006 and artemether–lumefantrine (AL) as second-line treatment in 2011 for uncomplicated falciparum malaria. Therapeutic efficacy studies conducted in 2011 and 2013 showed high AS + SP treatment failure rates (12–22%), with high levels of *Pfdhfr/Pfdhps* quadruple and quintuple mutations, while AL was highly efficacious [18, 19]. On the basis of this evidence, the NMCP recommended AL to replace AS + SP and DHA/PPQ as second-line treatment in 2016 [19]. The studies reported here were conducted to assess the therapeutic efficacy of DHA/PPQ and AL and to determine the mutations in *Pfk13* and amplification in the *Pfpm2* gene.

## Methods

### Study design, area and population

One-arm prospective studies were conducted with the standard WHO protocol [17] to evaluate clinical and parasitological responses to standard therapeutic doses of AL and DHA/PPQ in patients with uncomplicated falciparum malaria.

**Fig. 1:**
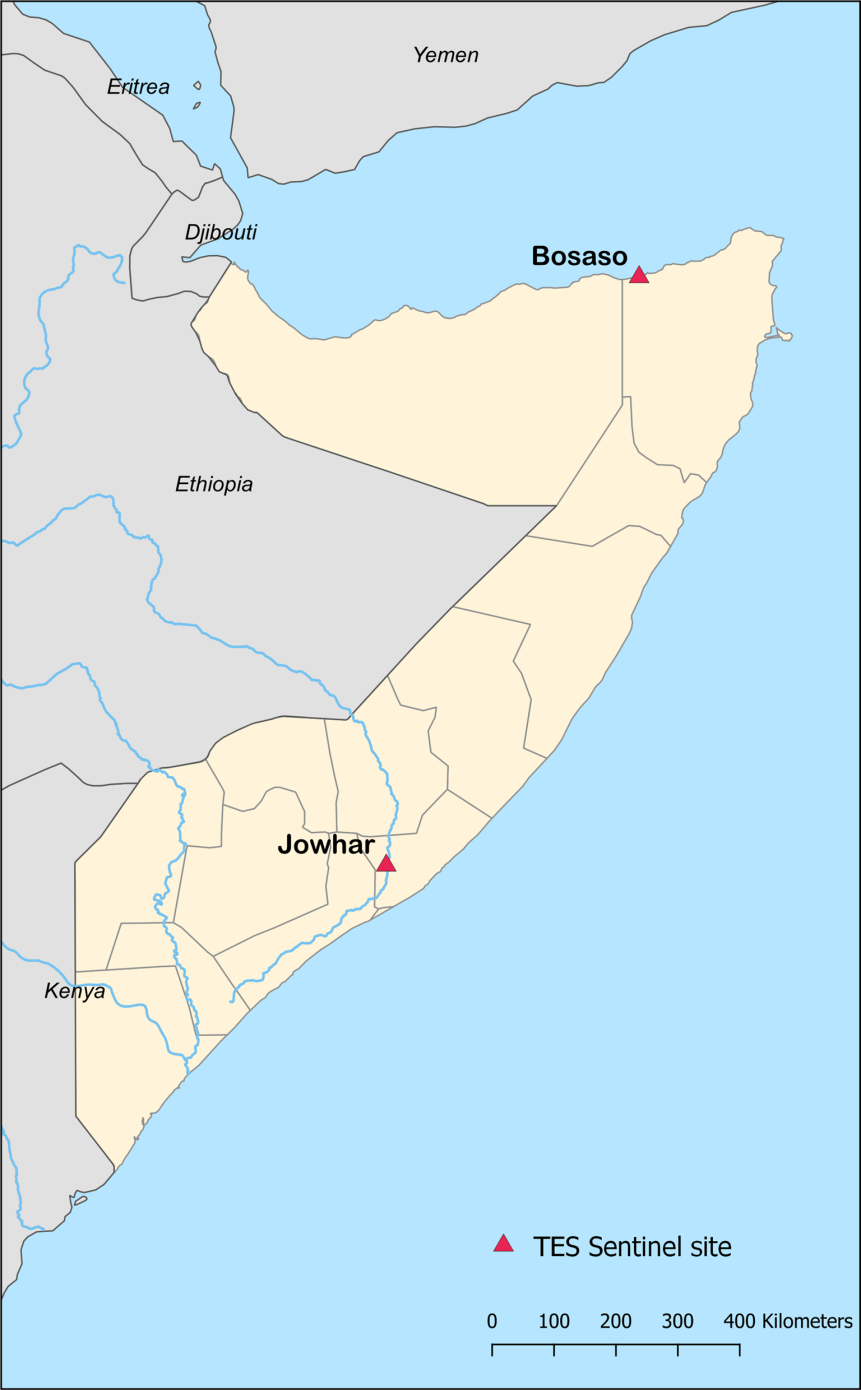
Map of Somalia showing the study sites (Jowhar and Bosaso)

The studies were conducted at two sentinel sites (Fig. 1): Jowhar in the Middle Shabelle region and Bosaso in the North-east region (Puntland). Previous studies reported moderate and low malaria transmission in Middle Shabelle and North-east regions, respectively [20, 21]. In Bosaso district, very few malaria cases (0/970–2/949; 0–0.2%) were recorded through the HMIS before the 2013 outbreak, when the reported number of confirmed malaria cases reached 19% (NMCP, unpublished data). A mobile clinic was set up in Jowhar town to recruit patients from rural communities within 10 km. In Bosaso, patients were recruited at the out-patient department of the regional hospital. The studies were conducted during the malaria transmission season at both sites.

Patients aged ≥ 6 months, excluding girls aged 12–17 years and unmarried women aged ≥ 18 years, as the local customs and culture would not allow pregnancy testing), with suspected uncomplicated malaria infection (axillary temperature ≥ 37.5 °C or history of fever during the previous 24 h) were tested for malaria parasites and were recruited if they had *P. falciparum* mono-infection with a parasite density of 500–200,000 asexual parasites/μL of blood and consented to participate in the study. There was no upper age limit. Patients were not enrolled if they presented with the following: severe malaria, mixed or mono-infection with non-falciparum species, known hypersensitivity to the study drugs, severe malnutrition, non-malaria febrile illness (measles, acute lower respiratory tract infection, severe diarrhoea with dehydration) or known underlying chronic diseases (e.g. cardiac, renal or hepatic disease, HIV/AIDS). In addition, patients on regular medication that might have interfered with the pharmacokinetics of the study ACT and those with a history of hypersensitivity reactions to the medicines were excluded.

### Treatment and follow-up

Eligible patients were given a standard therapeutic oral dose of either AL (Artemether–lumefantrine, IPCA Laboratories India) twice daily for 3 days or DHA/PPQ (Duo-Cortecxin^®^, Holley-Cotec Pharmaceuticals China) once daily for 3 days. AL (20/120 mg) was administered according to the following weight ranges: one tablet was given to patient weighing 5–14 kg; two tablets for 15–24 kg; three tablets for 25–34 kg and four tablets for ≥ 35 kg. DHA/PPQ was given as follows: half, three-quarters, one tablet, two tablets, three tablets and four tablets to patients weighing 5 to < 8 kg, 8 to < 14 kg, 14 to < 25 kg, 25 to < 36 kg, 36 to < 60 kg and 60 to < 80 kg, respectively. The study medicines were provided by the WHO, and all treatment doses were given under the direct supervision of a designated study team member. Each patient was observed for 30 min after administration; if he or she vomited during this period, the full dose was given again. Patients who vomited a second time were withdrawn from the study and referred or admitted to hospital, where parenteral artesunate was administered according to the national treatment policy.

Patients were followed up daily for the first 3 days after the first dose (day 0) and then weekly from day 7 onwards, up to day 28 for the AL-treated groups. Follow-up was extended to day 42 for DHA/PPQ-treated groups because piperaquine has a longer half-life compared to lumefantrine. Patients were also assessed on an unscheduled day if symptoms occurred. Clinical and laboratory evaluations were undertaken during the follow-up visits. Adverse events and severe adverse events, defined according to the WHO protocol for monitoring therapeutic efficacy of anti-malarial medicines [18], were monitored clinically at each follow‐up visit.

### Laboratory analysis

#### Microscopy

Thick and thin blood smears were taken from finger-prick blood on day 0 and during follow-up visits and stained with Giemsa. Parasites were counted on Giemsa-stained thick films and recorded as the number of asexual parasites per 200 white blood cells (or per 500, if the count was < 100 parasites/200 white blood cells). A smear was declared negative if no parasites were seen after 1000 white blood cells were counted. The presence of gametocytes at enrolment or follow-up days was recorded. In addition, 100 fields of the thick smear on day 0 were examined to exclude mixed infections; in case of any doubt, the thin film was examined for confirmation. Each blood smear was examined independently by two qualified microscopists, and parasite density (per µL) was calculated on the assumption of a leucocyte count of 8000 per µL blood. Final parasitaemia was calculated by averaging the readings of the two microscopists if they were in agreement (difference in parasite densities < 50%). If the two counts were discordant in terms of parasite positivity, species or density by > 50%, a third, independent microscopist re-examined the blood slides. For parasite species and positivity, two concordant results were considered the final result, while for parasite density, the average of the two closest estimates of parasitaemia was considered final.

#### Parasite genotyping

In order to differentiate recrudescence from re-infection, blood samples were collected on filter paper from a finger-prick on day 0 and on the day of parasite recurrence (day 7 onwards) for genotyping. Specimens were dried, stored in individual plastic bags with desiccants and protected from light, humidity and extreme temperatures. The samples were analysed at the Institut Pasteur, Cambodia. Each dried blood spot was punched with a sterile puncher, and the spots were placed in a 96-well plate in numerical order. Samples were lysed overnight in a saponin solution, and then DNA was extracted with Instagen Matrix resin, as previously described [22]. DNA samples (day 0 and day of recurrence) were analysed to genotype the highly polymorphic regions *msp1*, *msp2* (merozoite surface proteins 1 and 2) and *glurp* (glutamate-rich protein) loci, as recommended by the WHO [23]. The results were classified as recrudescence if the recurrent parasites were of the same parasite strain as those on day 0, or as a new infection if they were a different strain.

### Molecular markers

Parasite DNA was analysed on day 0 for the presence of mutations in the *Pfk13* propeller domain, which is associated with artemisinin resistance. The propeller domain was amplified in a nested-PCR assay, amplicons were sequenced according to Sanger’s method (Macrogen, Republic of Korea), and DNA sequences were analysed to identify specific single nucleotide polymorphisms (SNPs) related to artemisinin resistance [9]. The amino acid sequences were compared with the 3D7 wild-type amino acid sequences PF3D7_1343700. The presence of SNPs was confirmed by reading both the forward and the reverse strands. Parasites with mixed alleles were considered mutants. The number of copies of *Pfpm2* genes was assessed by reverse transcriptase-PCR (sybr green dye). The full method has been described by Witkowski et al. [10].

### Classification of treatment outcome

According to the WHO criteria [17], treatment responses were classified as early treatment failure (ETF), late clinical failure (LCF), late parasitological failure (LPF) or adequate clinical and parasitological response (ACPR) before and after protein Polymerase Chain Reaction (PCR) correction [17]. The primary end point was PCR corrected ACPR on day 28 for AL and day 42 for DP. Other outcomes were loss to follow-up and withdrawals, which included protocol violation, withdrawal of consent, failure to complete study treatment (persistent vomiting, self- or third-party administration of anti-malarial drug or antibiotics with anti-malarial activity, occurrence during follow-up of concomitant disease that would interfere with a clear classification of the treatment outcome; detection of mono-infection with another malaria species during follow-up, or misclassification of a patient due to a laboratory error (parasitaemia) leading to administration of rescue treatment.

### Sample size

Assuming a treatment failure of 5% for both AL and DHA/PPQ with 95% confidence level and 5% precision, a minimum sample of 73 patients per site per drug was estimated. With a 20% increase to allow loss to follow-up and withdrawals during the 28-day (AL) or 42-day (DHA/PPQ) follow-up period, 88 patients per site per drug were targeted.

### Statistical analysis

The clinical and laboratory data of each patient were recorded on standard case record forms, double-entered independently and analysed with an WHO Excel^®^ database specifically designed for studies of anti-malarial drug efficacy (https://www.who.int/malaria/areas/drug_resistance/efficacy-monitoring-tools/en/).

This software automatically computes treatment outcomes by both per-protocol and Kaplan–Meier analysis. For the per-protocol analysis, patients were excluded if they were lost to follow-up or withdrawn because of withdrawal and protocol violation. In addition, patients with reinfections or undetermined or missing PCR were also excluded from PCR corrected per-protocol analysis. For the Kaplan–Meier analysis, patients who were lost to follow-up or withdrawn were censored on the last day of follow-up according to the timetable. For the PCR corrected Kaplan–Meier analysis, patients with reinfection were censored on the day of reinfection while those with undetermined or missing PCR were totally excluded.

Baseline characteristics (age, gender, temperature, parasitaemia) at the two study sites were compared by study drug. Chi squared tests were used to compare categorical data. Differences in the baseline mean age and parasite density were evaluated in a two-sample Wilcoxon rank-sum (Mann–Whitney) test for non-normally distributed data. Gametocyte positivity rates on enrolment and on follow-up days were computed. In addition, clearance of gametocytaemia after treatment among gametocyte carriers at recruitment was assessed by Kaplan–Meier survival analysis. Confidence intervals were calculated for binomial proportions.

### Ethical considerations

The study protocol was approved by the Ministry of Health of the Federal Government of Somalia, the Ministry of Health of Puntland and the WHO Research Ethics Review Committee. Patients or parents/guardians of study children were informed about the study and provided written consent. If a patient, parent or guardian was illiterate, he or she chose a witness to co-sign the consent form. Informed assent was obtained from children aged ≥ 12 years. Travel costs for scheduled and unscheduled visits were reimbursed. In Jowhar, community leaders of the study villages were informed about the study objectives and procedures and gave their permission.

## Results

### Baseline characteristics

**Fig. 2:**
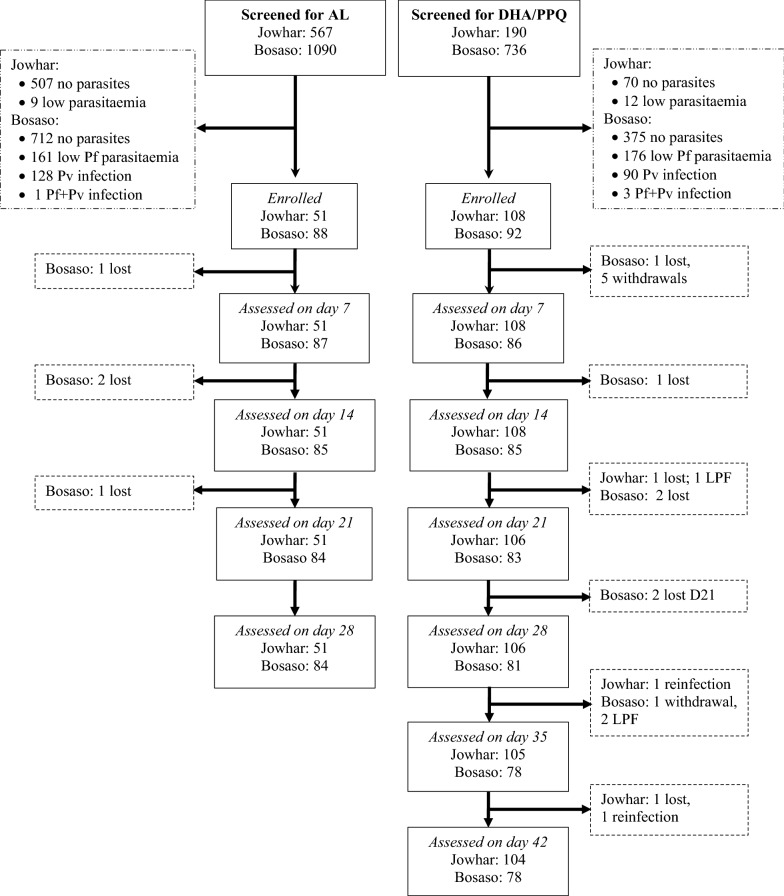
Patient flow chart for AL and DHA/PPQ cohorts in Jowhar and Bosaso sites. AL, artemether–lumefantrine; DHA/PPQ, dihydroartemisinin–piperaquine; Pf, *Plasmodium falciparum*; Pv, *Plasmodium vivax*

Potential study patients were screened in Jowhar from August to October 2016 (DHA/PPQ) and 2017 (AL), and in Bosaso from January to February (DHA/PPQ), and from November 2017 to February 2018 (referred as 2017 in the rest of the document) for AL. A total of 2583 febrile patients were screened for eligibility criteria and 339 patients were recruited (Fig. 2). Out of these, 200 patients (92 in Bosaso, 108 in Jowhar) were treated with DHA–PPQ and 139 (88 in Bosaso, 51 in Jowhar) with AL. The target sample of 88 patients could not be reached in Jowhar in 2017 because of the lower parasite positivity rate among screened febrile patients during the transmission season of 2017 (10.6%) than in 2016 (63.2%).

The baseline age, axillary temperature and parasitaemia of the study patients are summarized in Table 1. Significantly more males than females were recruited at both sites, which was expected, as girls aged 12–17 years and unmarried women aged ≥ 18 years were not enrolled. The mean age of patients in Bosaso was significantly (*P *< 0.0001) higher than that in Jowhar for both DHA/PPQ and AL groups. The geometric mean parasite density at enrolment was higher among patients in Jowhar than in Bosaso, although the difference was significant (*P *< 0.01) only between the groups treated with DHA/PPQ. The proportion of patients with detectable gametocytaemia at enrolment was higher among patients in Jowhar than in Bosaso for both treatment groups (Table 1), but the difference was significant only between the DHA/PPQ groups (*X*^*2*^= 13.9; *P* < 0.001) and not for the AL groups (*X*^*2*^ = 3.6; *P* = 0.06).

**Table 1:** Baseline characteristics of study patients in Bosaso and Jowhar sites, Somalia

Characteristic	DHA–PPQ (2016)		AL (2017)	
	Bosaso (n = 92)	Jowhar (n = 108)	Bosaso (n = 88)	Jowhar (n = 51)
Males, n (%)	83 (90.2)	76 (70.4)	65 (73.9)	41 (80.4)
Mean age years (SD)^a^	25 (14.6)	9.7 (6.5)	17 (10.4)	10.2 (6)
Age group, n (%)				
< 5 years	8 (8.7)	15 (13.9)	4 (4.5)	5 (9.8)
5 to 15 years	19 (20.6)	83 (76.8)	38 (43.2)	39 (76.5)
≥ 15 (adults)	65 (70.7)	10 (9.3)	46 (52.3)	7 (13.7)
Axillary temperature (°C)				
Mean (SD)	38.2 (0.3)	38.0 (0.5)	38 (0.4)	38 (0.5)
Parasitaemia (per µL)				
Geometric mean^b^	7466	11,074	8042	10,454
Range (min–max)	1300–74,768	597–144,000	811–83,163	1012–79,196
Gametocytaemia, n (%)	9 (9.8)	34 (31.5)	10 (11.4)	12 (23.5)

SD, standard deviation

^a^The mean age of the Bosaso study population was significantly (P < 0.0001) greater than those of Jowhar for both AL and DP treatment groups

^b^Difference between the parasite density of Jowhar and Bosaso was significant (P < 0.02)

### Treatment outcomes

Eight and six patients were lost to follow and withdrawn, respectively (Fig. 2). The reasons for withdrawal included death (n = 1), withdrawal of consent (n = 2), imprisonment (n = 1) and third-party treatment with antimalarial drug (n = 2). Table 2 summarizes the treatment outcomes at 28-days and 42-days of follow-up for AL and DHA/PPQ, respectively. In the DHA/PPQ-treated groups, five patients (two in Bosaso and three in Jowhar) showed parasite recurrence on day 14 (n = 1), day 28 (n = 3) and day 35 (n = 1). Only one of the three patients in Jowhar carried recrudescent parasites, while the other two cases were re-infections, giving a PCR-corrected ACPR rate of 99.0% (95% CI, 92.0%–99.4%). The samples from the two patients in Bosaso were not corrected by PCR because suitable samples were not available at the time of recurrence. Even in the worst-case scenario (both patients with recrudescence), however, the outcome would have led to an ACPR rate of 97.5% (95% CI 91.3%–99.7%). No parasite recurrence was observed in the AL-treated groups giving an ACPR rate of 100% (95% CI 93%–100%) in Jowhar and 100% (95% CI 95.7%–100%) in Bosaso (Table 2). All patients in both AL and DHA/PPQ treated groups were parasite-free on day 3, as verified by microscopy.

**Table 2:** Treatment outcome in patients with uncomplicated falciparum infection treated with dihydroartemisinin–piperaquine (DHA/PPQ) or artemether–lumefantrine (AL) in Bosaso and Jowhar sites, Somalia

Treatment responses	DHA-PP		AL	
	Bosaso (N = 92)	Jowhar (N = 108)	Bosaso (N = 88)	Jowhar (N = 51)
Parasitaemia on day 3	0 (0)	0 (0)	0 (0)	0 (0)
PCR-uncorrected				
ETF, n (%)	0 (0)	0 (0)	0 (0)	0 (0)
LCF, n (%)	0 (0)	0 (0)	0 (0)	0 (0)
LPF, n (%)	2 (2.5)	3 (2.8)	0 (0)	0 (0)
ACPR, n (%)	78 (97.5)	103 (97.2)	84 (100)	51 (100)
Total per protocol	80	106	84	51
Lost/withdrawn, n (%)	12 (13.0)	2 (1.9)	4 (4.5)	0 (0)
Kaplan–Meier: cure rate	78 (97.5)	103 (97.2)	84 (100)	51 (100)
PCR-corrected				
ETF, n (%)	0 (0)	0 (0)	0 (0)	0 (0)
LCF, n (%)	0 (0)	0 (0)	0 (0)	0 (0)
LPF, n (%)	NA*	1 (1.0)	0 (0)	0 (0)
ACPR, n (%)	NA*	103 (99.0)	84 (100)	51 (100)
Total per protocol	NA*	104	84	51
Lost/withdrawals/re-infection: n (%)	NA*	4 (3.7)	4 (4.5)	0 (0)
Kaplan–Meier: cure rate	NA*	103 (99.1)	84 (100)	51 (100)

*PCR analysis to differentiate between recrudescence and new infection was not done for the two patients in the DHA/PPQ group in Bosaso because suitable samples were not available at the time of recurrence

ETF, early treatment failure; LCF, late clinical failure; LPF, late parasitological failure; ACPR, adequate clinical and parasitological response

**Fig. 3:**
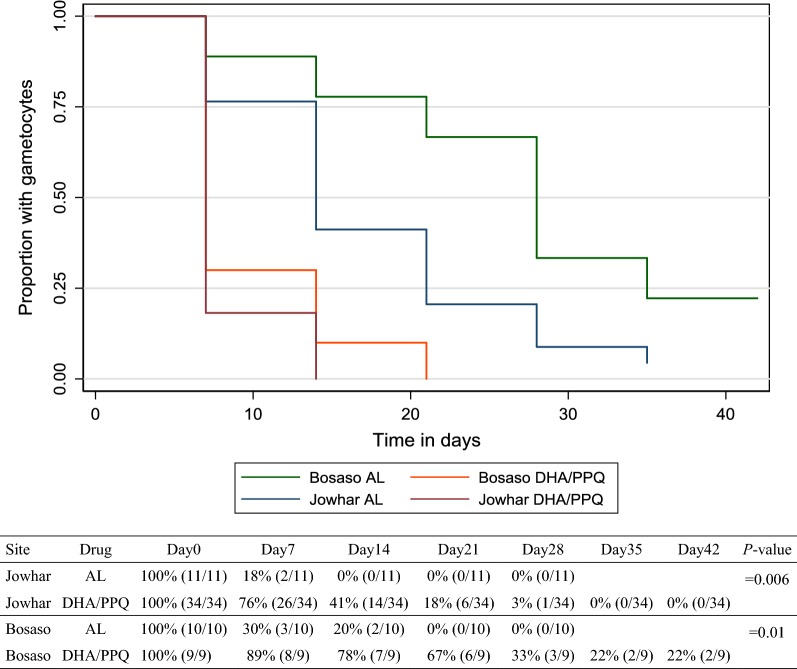
Gametocyte clearance after treatment evaluated in patients with gametocytaemia at recruitment

Of the patients with detectable gametocytaemia at enrolment, clearance of gametocytaemia was significantly faster with AL than with DP in Jowhar (HR: 3.0, 95% CI 1.4–6.5; *P *= 0.006) and in Bosaso (HR: 6.1, 95% CI 1.5–24.3; *P *= 0.01), as shown in Fig. 3. Only three and four patients who had no gametocytes at enrolment developed gametocytaemia after treatment AL and DHA/PPQ treatment, respectively.

### Adverse events

One serious adverse event (death of a child) was recorded but it was not related to the study drug but to a car accident. The child, a girl aged 11 years, presented with an axillary temperature ≥ 38.2 °C and a parasite density of 44,466 asexual parasites/μL. She completed medication, and no parasites were detected on days 2 and 3 but did not attend the day 7 visit. Otherwise, no other adverse event was observed, and the drug was well tolerated.

### PfK13 and PfPM2

Day-0 samples from the 139 patients in the AL study were tested for the presence of polymorphism in the *Pfk13* gene and amplification of *Pfpm2* genes. For the *Pfk13* analysis, 138 samples gave interpretable results, and only one sample (0.7%) from Bosaso showed a non-synonymous mutation at codon 622 (R622I). Among the 139 samples analysed for *Pfpm2* gene amplification, 135 gave interpretable results, and none showed *PfPM2* amplification.

## Discussion

The findings of the studies demonstrate that AL and DHA/PPQ are highly effective, supporting the recent recommendation of the Somalia NMCP for use of these artemisinin-based combinations as first-line and second-line treatments for uncomplicated malaria in Somalia. With respect to artemisinin resistance, none of the patients was parasitaemic on day 3 after either DHA/PPQ or AL, indicating an adequate response of the parasites to the artemisinin component. *Pfk13* non-synonymous mutations (R622I) were present at a very low rate (1/138). The impact of this mutation on the parasite resistance phenotype is currently unknown, and it has also been detected in other locations in East Africa, such as Ethiopia [24]. Additional investigations, with in vitro testing of parasites carrying the R622I mutation are, therefore, essential to determine the extent to which circulation of this allele in East Africa is a threat.

The very high ACPR rate with both artemisinin-based combinations indicates excellent therapeutic activity of both partner drugs. This was confirmed genetically for PPQ by the absence of *Pfpm2* amplification in the parasites collected from the study areas. The same analysis could not be conducted for lumefantrine resistance because of the absence of reliable molecular markers.

AL, the most commonly recommended ACT medicine in malaria-endemic countries, remains highly efficacious in Africa, despite its use as first-line treatment of uncomplicated falciparum malaria for more than a decade [25–41]. DHA/PPQ has recently been adopted as second-line treatment in several malaria-endemic countries in the African and Eastern Mediterranean regions, and the available data show high efficacy [25, 27, 30, 34–37, 39, 41, 42]. This high efficacy may be explained by the recent introduction of DHA/PPQ as second-line treatment, suggesting weak drug pressure through its limited use. Previous experience with use of DHA/PPQ in countries in the Greater Mekong sub-region nevertheless shows that, initial high efficacy (96–98%) [43, 44] can decrease rapidly [7, 8, 13, 45]. In Cambodia, DHA/PPQ was recommended as first-line treatment in 2008 in Pailin Province and in 2010 for the whole country [3] because of unacceptably high treatment failure with artesunate–mefloquine [46]. This was immediately followed by a substantial decrease in the efficacy of DHA/PPQ in Pailin (from 89% in 2008–2009 to 75% in 2010–2011) [47]. A study in 2012–2013 confirmed the rapid decrease in treatment efficacy and its spread to other provinces of Cambodia [13]. Recent studies have shown that DHA/PPQ treatment failure is associated with resistance to PPQ [13, 45]. Similarly, an increase in the DHA/PPQ treatment failure rate from 0 to 26% was found over 3 years in Viet Nam, with a background of increased molecular markers for artemisinin and PPQ resistance [7], as confirmed by Phuc et al. [8]. The explanation for this unprecedented, generalized failure of an ACT was later provided by demonstration of parasites that are multi-resistant to DHA and PPQ [8] and their spread in the Greater Mekong sub-region [48]. The reason for the rapid emergence of DHA/PPQ resistance in this sub-region is unknown; however, pre-existing circulation of parasites resistant to artemisinin or PPQ in this region was certainly a major facilitator for their evolution to multidrug resistance. This history of rapid resistance selection calls for combining any DHA/PPQ implementation initiative with regular, close clinical and biological monitoring of resistance.

The post-treatment rate of gametocytaemia clearance varied significantly between AL and DP treated groups, with DHA/PPQ being less potent, confirming previous reports on slower gametocyte clearance with DHA/PPQ treatment than with AL [49–54]. The cause of the differential effect of DHA/PPQ and AL on clearance of gametocytaemia is not clear but might be linked to the different partner drug activity.

## Conclusions

The findings of these studies document high efficacy of AL and DHA/PPQ for the treatment of uncomplicated falciparum malaria as well as lack of evidence of resistance to artemisinins and PPQ, showing the relevance of these two combinations in the chemotherapeutic strategy for malaria control in Somalia. Regular monitoring of the efficacy of these artemisinin-based combinations and of markers of resistance for artemisinin and partner drugs should be continued in order to generate evidence to inform national malaria treatment policies.

## Data Availability

The datasets generated and analysed during the current studies have been shared with WHO to contribute to the WHO database.

## Abbreviations

ACPR: adequate clinical and parasitological response
ACT: artemisinin-based combination therapy
AL: artemether–lumefantrine
DHA/PPQ: dihydroartemisinin–piperaquine
*glurp*: glutamate-rich protein gene
NMCP: National Malaria Control Programme
*msp1*: merozoite surface protein 1 gene
*msp2*: merozoite surface protein 2 gene
*Pfk13*: *Plasmodium falciparum Kelch 13 gene*
*Pfpm2*: *Plasmodium falciparum* plasmepsin2 gene
WHO: World Health Organization
